# Selected Papers from ENZITEC 2010

**DOI:** 10.4061/2011/863045

**Published:** 2011-12-29

**Authors:** Denise M. G. Freire, Elba P. S. Bon, Gustavo Viniegra-Gonzalez, Francisco Girio, Robert F. H. Dekker

**Affiliations:** ^1^Laboratory of Microbial Biotechnology, Department of Biochemistry, Institute of Chemistry, Federal University of Rio de Janeiro, Avenida Athos da Silveira Ramos 149, Centro de Tecnologia, Bloco A, Ilha do Fundão, 21941-909 Rio de Janeiro, RJ, Brazil; ^2^Laboratory of Enzyme Technology, Department of Biochemistry, Institute of Chemistry, Federal University of Rio de Janeiro, Avenida Athos da Silveira Ramos 149, Centro de Tecnologia, Bloco A, Ilha do Fundão, 21941-909 Rio de Janeiro, RJ, Brazil; ^3^Departamento de Biotecnología, Universidad Autónoma Metropolitana, 09340 Iztapalapa, DF, Mexico; ^4^Laboratório Nacional de Energia e Geologia, I.P., Unidade de Bioenergia, Edifício F, Estrada do Paço do Lumiar 22, 1649-038 Lisbon, Portugal; ^5^Biorefining Research Initiative, Lakehead University, 955 Oliver Road, Thunder Bay, ON, Canada P7B 5E1

The fundamental need for environmental preservation has been calling, around the world, for the development and deployment of friendly industrial processes. Moreover, presently, development has to take into account sustainability. Within this context, enzyme technology is of outmost importance, having a major role for the achievement of the goals of sustainable development. This technology is of particular significance to Brazil due to the need to preserve Brazil's singular ecosystems, biodiversity, and quality and availability of the country's water resources. Furthermore, Brazil has an unrivalled availability of natural wealth, in both diversity and quantity, to be processed, via biocatalysis, into a wide range of diverse and innovative products as well as into biofules. Enzymes are the logical tool to process renewable resources as both of them have naturally evolved to match each other. It is now, therefore, time to develop dexterity and expand the use of enzymes in a fully efficient manner. As biochemical processes are cleaner than its chemical counterparts and generate higher-quality products, this shift would benefit the country's socioeconomic development. 

However, going from traditional chemistry into enzymatic processes is more than a technical and economical challenge, it is a change in the way we think technology—scientific and technical knowledge— and thoughtfulness are much needed. 

The Brazilian Seminar on Enzyme Technology (ENZITEC) has been taking place every two years since 2003. Its main objective has been to further the knowledge on the wide range of industrial, technical, and specialty enzymes alongside promoting its economic viability. The event encompasses the worldwide interest in industrial enzyme research and uses *vis-à-vis* the necessary move toward biocatalysis. Eight papers, which were selected from a wealth of submitted manuscripts, were accepted for publication in this special issue of Enzyme Research. They relate to fundamental areas of enzyme research and technology such as microbial screening, enzymes production and characterization, fine chemistry, biorefinery, and biofuels. 

*Denise M. G. Freire* *Denise M. G. Freire* *Elba P. S. Bon* *Elba P. S. Bon* *Gustavo Viniegra-Gonzalez* *Gustavo Viniegra-Gonzalez* *Francisco Girio* *Francisco Girio* *Robert F. H. Dekker* *Robert F. H. Dekker*

